# Comparison of Resting-State Brain Activation Detected by BOLD, Blood Volume and Blood Flow

**DOI:** 10.3389/fnhum.2018.00443

**Published:** 2018-11-08

**Authors:** Ke Zhang, Dengfeng Huang, N. Jon Shah

**Affiliations:** ^1^Institute of Neuroscience and Medicine INM-4, Medical Imaging Physics, Forschungszentrum Jülich, Jülich, Germany; ^2^Department of Neurology, Faculty of Medicine, JARA, RWTH Aachen University, Aachen, Germany

**Keywords:** resting-state fMRI, brain networks, cerebral blood flow, cerebral blood volume, VASO, ASL, BOLD

## Abstract

Resting-state brain activity has been widely investigated using blood oxygenation level dependent (BOLD) contrast techniques. However, BOLD signal changes reflect a combination of the effects of cerebral blood flow (CBF), cerebral blood volume (CBV), as well as the cerebral metabolic rate of oxygen (CMRO_2_). In this study, resting-state brain activation was detected and compared using the following techniques: (a) BOLD, using a gradient-echo echo planar imaging (GE-EPI) sequence; (b) CBV-weighted signal, acquired using gradient and spin echo (GRASE) based vascular space occupancy (VASO); and (c) CBF, using pseudo-continuous arterial spin labeling (pCASL). Reliable brain networks were detected using VASO and ASL, including sensorimotor, auditory, primary visual, higher visual, default mode, salience and left/right executive control networks. Differences between the resting-state activation detected with ASL, VASO and BOLD could potentially be due to the different temporal signal-to-noise ratio (tSNR) and the short post-labeling delay (PLD) in ASL, along with differences in the spin-echo readout of VASO. It is also possible that the dynamics of spontaneous fluctuations in BOLD, CBV and CBF could differ due to biological reasons, according to their location within the brain.

## Introduction

The measurement of functional connectivity (FC) in the resting state has been a powerful method with which to characterize the intrinsic functional architecture of the brain (Biswal et al., 1995; Greicius et al., 2003; Fox et al., 2005). It has been suggested that FC may reflect relevant task-induced activation and behavioral performance (Hampson et al., 2006; Zou et al., 2013), and could be a biomarker for diagnosing diseases and for the monitoring of treatment outcomes (Buckner et al., 2008; Fornito and Bullmore, 2010; Salvador et al., 2010; Menon, 2011; Miao et al., 2014). It is possible to acquire functional signals using magnetic resonance imaging (MRI) based on blood oxygenation level dependent (BOLD) contrast, cerebral blood flow (CBF), cerebral blood volume (CBV) and cerebral metabolic rate of oxygen (CMRO_2_).

Among these techniques, resting-state BOLD fMRI is the most widely used. This is due to its simple MRI acquisition sequence, high sensitivity and relatively high spatial and temporal resolution. However, BOLD signal changes reflect a combination of effects from blood oxygenation, CBV, CBF and CMRO_2_ (Davis et al., 1998; Hoge et al., 1999; Buxton et al., 2004). In contrast, imaging techniques based on the physiological parameters of CBV, CBF or CMRO_2_ are collected with fewer confounds and are straightforward to interpret. In addition, although the literature relating to BOLD-based FC is extensive, it is relatively disorganized and there is proportionately very little relating to CBF-CBV- and CMRO2-based FC. A non-BOLD approach is therefore highly desirable in order to clarify the meaning of the BOLD-based FC literature.

It has been widely demonstrated that CBF is coupled with BOLD, which has also been shown to be linked to brain metabolism (Raichle, 1998). This is the case in both active and resting states (Fox and Raichle, 1986; Fox et al., 1988; Raichle et al., 2001; Jann et al., 2015; Storti et al., 2017; Chiacchiaretta et al., 2018). Changes in CBF can be measured non-invasively with arterial spin labeling (ASL; Barbier et al., 2001; Golay et al., 2004). In ASL, the perfusion contrast in the images is generated by the subtraction of successively acquired images: one with and one without the proximal labeling of arterial spins after a delay time. The subtracted signal is on the order of 1% of the baseline signal, and resting-state fluctuations cause merely an additional fractional change. The main challenges in using ASL to observe resting-state CBF fluctuations are the low signal-to-noise ratio (SNR), low temporal resolution and possible contamination from BOLD fluctuations. Continuous ASL imaging, which provides higher SNR compared to pulsed ASL, has been used to investigate resting-state brain activity as a comparison with BOLD (Chuang et al., 2008; Viviani et al., 2011; Li et al., 2012). High-pass filtering of the ASL signal allows for CBF oscillations to be isolated with reduced BOLD contamination (Chuang et al., 2008). Connectivity maps from the CBF and the BOLD signal have been demonstrated to be regionally similar (Fukunaga et al., 2008; Viviani et al., 2011; Li et al., 2012; Chen et al., 2015).

The use of the spontaneous fluctuation of the CBV-weighted signal using whole-brain gradient and spin echo (GRASE) based vascular space occupancy (VASO) to detect resting-state networks has also been demonstrated (Miao et al., 2014). The VASO technique makes use of the T_1_ difference between blood and the surrounding brain tissue and uses an inversion recovery pulse sequence to null the blood signal while maintaining part of the tissue signal. The VASO signal intensity is thus proportional to 1-CBV. When neural activation causes the CBV to increase, the VASO signal shows a decrease, allowing the detection of activated regions in the brain (Lu and van Zijl, 2012). Using 3D-GRASE as a readout method, the data from the entire brain can be collected at the blood nulling time. This implies the possibility of observing changes in CBV in the entire brain (Gunther et al., 2005; Poser and Norris, 2009; Miao et al., 2014). Reliable brain networks were detected from the CBV-based, whole-brain images, including the default mode network, the salience, the executive control, the visual, the auditory and the sensorimotor networks. The improved spatial location offered by the VASO technique, compared to BOLD, was shown in task-evoked brain activation, in which activation aligned well with gray matter in VASO but extended to other areas in BOLD. Moreover, 3D-GRASE VASO images showed less sensitivity to susceptibility artifacts.

Great effort has been devoted to the comparison of BOLD, CBV and CBF (Chen and Pike, 2009; Wu et al., 2009; Shih et al., 2011; Sforazzini et al., 2014; Krieger et al., 2015; Donahue et al., 2017), and comparisons between BOLD, CBV and CBF have been performed in visual and in sensorimotor stimulations in human studies (Chen and Pike, 2009; Krieger et al., 2015). Resting-state FC maps from the CMRO_2_ signal are, in general, similar to those from BOLD and perfusion in human studies (Wu et al., 2009). Multimodal functional imaging was also performed in animal models for the study of pain (Shih et al., 2011). It was found that BOLD and CBV contrast produced consistent resting-state networks in mouse models (Sforazzini et al., 2014). However, thus far, no comparison of resting-state networks has been reported in relation to the combined use of BOLD, CBV and CBF in human studies. In this work, resting-state brain activation, detected by three contrasts including BOLD, CBV and CBF, were compared in order to investigate/observe differences in: (a) seed-based FC; (b) temporal and frequency characteristics; and (c) FC matrices.

## Materials and Methods

### Subjects

Sixteen healthy subjects (eight male, eight female; mean age 28 ± 5 years) were scanned using a 32-channel head receive RF coil on a 3T Trio Siemens scanner (Siemens Healthcare, Erlangen, Germany). All subjects were highly educated, relatively young, healthy volunteers (20 < age < 40 years). Participants were excluded if they suffered from a neurological or psychiatric illness, any untreated medical illness, such as uncontrolled diabetes or treatment-resistant hypertension, or if they had any metallic implants. Written informed consent was obtained from all subjects and the study was approved by the Ethics Committee of the Medicine Faculty of the Rheinisch-Westfälischen Technischen Hochschule Aachen (RWTH Aachen University). The study was conducted in accordance with the Declaration of Helsinki. The order of BOLD, VASO and ASL scans was pseudo-randomized among participants. The subjects were instructed to close their eyes and to refrain from falling asleep during the scans.

### Image Acquisition

A gradient-echo echo planar imaging (GE-EPI) sequence was used for BOLD imaging acquisition. The sequence parameters of the EPI measurement were: α/TE/TR = 90°/30/2,500 ms, matrix = 64 × 64 × 36, resolution = 3.4 × 3.4 × 3 mm^3^. Each BOLD measurement included 130 repetitions and was performed in 5.4 min.

A VASO sequence with a global inversion pulse, for the purpose of blood nulling and a single-shot GRASE, for readout, was implemented. The specific sequence parameters of the 3D-GRASE based readout were: TI/TE/TR = 740/16.4/2,500 ms, flip angle = 180°, matrix = 64 × 64 × 24, resolution = 3.4 × 3.4 × 5 mm^3^, GRAPPA factor along ky = 3, partial Fourier along k_z_ = 6/8, bandwidth = 1,776 Hz/pixel and total readout length = 295. TI was determined based on the blood T1 of 1,627 ms (Lu et al., 2004; Poser and Norris, 2011; Miao et al., 2014). Each VASO measurement included 130 repetitions and was performed in 5.4 min.

For ASL-MRI, a pseudo-continuous arterial spin labeling (pCASL) sequence was considered as it offers a high SNR (Wu et al., 2007; Dai et al., 2008). pCASL uses a 1 s train of RF and gradient pulses to invert the magnetization of blood water flowing through the labeling plane (Okell et al., 2010). In our experiments, the position of the labeling plane was selected from a quick time-of-flight angiography to ensure the optimal orientation of the carotid and vertebral arteries. The positioning of the labeling plane was located at the point where the main arteries run approximately in the inferior-superior direction, and twists in the arteries were avoided. Typically, a position between the two “twists” in the vertebral arteries, before they fuse to form the basilar artery, was chosen. A delay of 1,050 ms between labeling and readout was included to guarantee blood perfusion in the majority of the voxels. Pre-saturation pulses were applied to the imaging region before labeling to avoid the spin perturbation in imaging planes caused by the labeling train. By using readouts with single-shot 2D EPI, 130 measurements with 65 pairs of label-control volumes were obtained. Explicitly, sequence parameters were as follows: α/TE/TR = 90°/14/3,000 ms, dim: 64 × 64 × 20, Partial Fourier = 6/8, resolution: 3.4 × 3.4 × 5 mm^3^. The total measurement time for pCASL acquisitions was 7.6 min.

Finally, a conventional 3D MP-RAGE sequence of 6 min was performed to acquire T1-weighted anatomical images (resolution = 1 mm^3^ isotropic, TI/TR = 1,100/2,530 ms, flip angle = 7°, 256 × 256 × 176, GRAPPA factor = 2).

### Data Processing

#### Preprocessing

The resting-state BOLD, VASO and ASL were preprocessed using DPARSF^1^ with the following steps: slice-timing (only applied to BOLD), motion correction, spatial normalization to the standard Montreal Neurological Institute (MNI) space, smoothing along three directions with FWHM = 6 mm (CBF calculation), detrending and band-filtering (0.01–0.08 Hz) to remove certain nuisance covariates, including six head motion parameters, global mean signal, white matter signal and cerebrospinal fluid signal. The functional images were normalized to MNI space by using a unified segmentation on T1 to improve the accuracy of the spatial normalization. This procedure contains three steps: co-registration, segmentation and the writing of normalization parameters. To ensure that CBF values were not contaminated by the BOLD effect, spatially smoothed ASL raw data was split into a high-pass filtered series with a cut-off at 1/(4*TR) (Chuang et al., 2008), which was 0.08 Hz in our case. A low-pass series was obtained as a residual of the filtering. The high-pass filtering range applied to the ASL signal to generate uncontaminated CBF fluctuations was 0.08–0.2 Hz. The high-passed filtered series was used to obtain the CBF signal. The calculation of the CBF was obtained using ASLtbx (Wang et al., 2008) and a surround subtraction method was used on the high-pass filtered series. After CBF calculation, the CBF data were detrended and nuisance covariates were removed by regression, as was also the case for BOLD and VASO.

### Seed-Based Functional Connectivity Analyses

To obtain the corresponding networks in the resting-state, eight seed spherical regions-of-interest (ROIs), with radii of 6 mm, were placed in the left precentral gyrus (Talairach coordinates: −53, −7, 29), left transverse temporal gyrus (−50, −21, 11), left cuneus (BA17: −6, −76, 11), left inferior occipital gyrus (BA17: −20, −94, −8), left posterior cingulate cortex (−12, −54, 10), left dorsal cingulate cortex (−4, 26, 34), left inferior parietal cortex (−48, −63, 38) and right inferior parietal cortex (45, −58, 42). The cross-correlation coefficient (cc) maps for each subject were calculated based on the extracted average time course of each seed ROI. These maps were then transformed to z-value maps using Fisher’s z transform. A one-sample *t*-test was performed on these z-value maps to acquire significant FC maps at a group level, which were thresholded at *t* > 7.68, with a cluster size resulting in a *p*-value *p* < 0.05 (FWE). The numbers of active voxels and the summed *t* of these active voxels in different networks were compared.

### Time Courses and Spectra

To explore the frequency characteristic of the three contrasts, unfiltered resting-state BOLD, VASO and ASL time courses were extracted from seed ROIs in the primary visual cortex and default-mode network of all subjects, and their spectra were computed.

### Adjacency Matrix Construction

One hundred and sixteen network nodes were defined using the anatomical automatic labeling (AAL) atlas (Tzourio-Mazoyer et al., 2002). Subsequently, 116 region-wise, mean time-courses were employed to calculate the functional connection based on their Pearson’s correlation. This resulted in one (116 × 116) correlation matrix for each subject, representing interregional FC. The group-level connectivity matrix was averaged across the individual network matrix under BOLD, VASO and ASL. A one-sample *t*-test was performed to confirm the significance of connectivity in each group.

### Results

**Figure 1:**
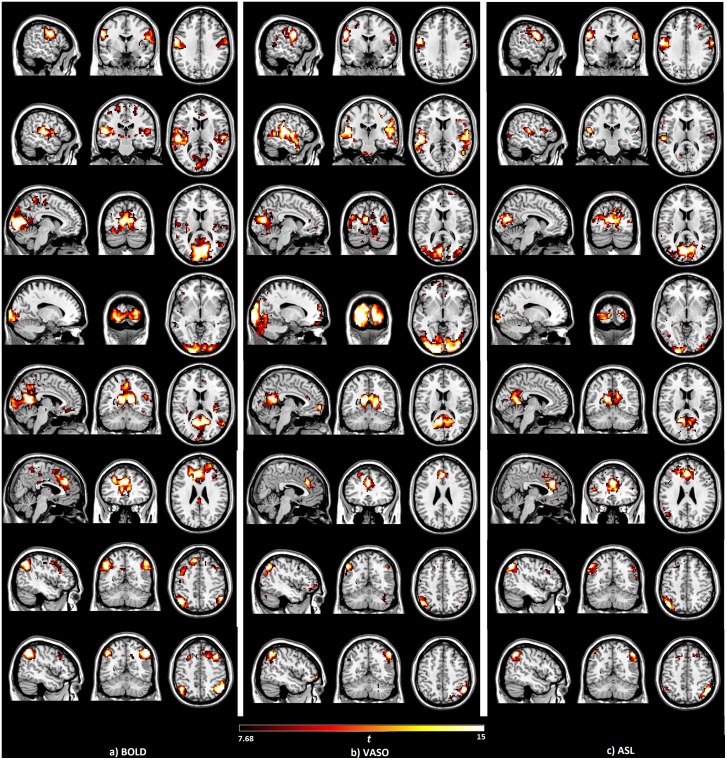
Brain networks detected by the seed-based analysis of the **(A)** blood oxygenation level dependent (BOLD), **(B)** vascular space occupancy (VASO) and **(C)** arterial spin labeling (ASL) data, including sensorimotor, auditory, primary visual, higher visual, default mode, salience and left/right executive control networks.

For comparison, the seed-based function networks generated from BOLD, VASO and ASL are displayed side by side in Figure 1. Eight resting functional networks were generated, which included the sensorimotor network, the auditory network, the primary visual network, the higher visual network, the default-mode network, the salience network, the left executive-control network (ECN) and the right ECN. The location and strength of the connectivities, based on their *t* values, are similar among all three contrasts. In all the networks observed, with the exception of the higher visual network, the connectivities based on BOLD were found to be higher than those of VASO and ASL. Most of the networks show higher connectivity in VASO than in ASL. Compared to VASO, the sensorimotor and the salience network in ASL had higher connectivities.

**Figure 2:**
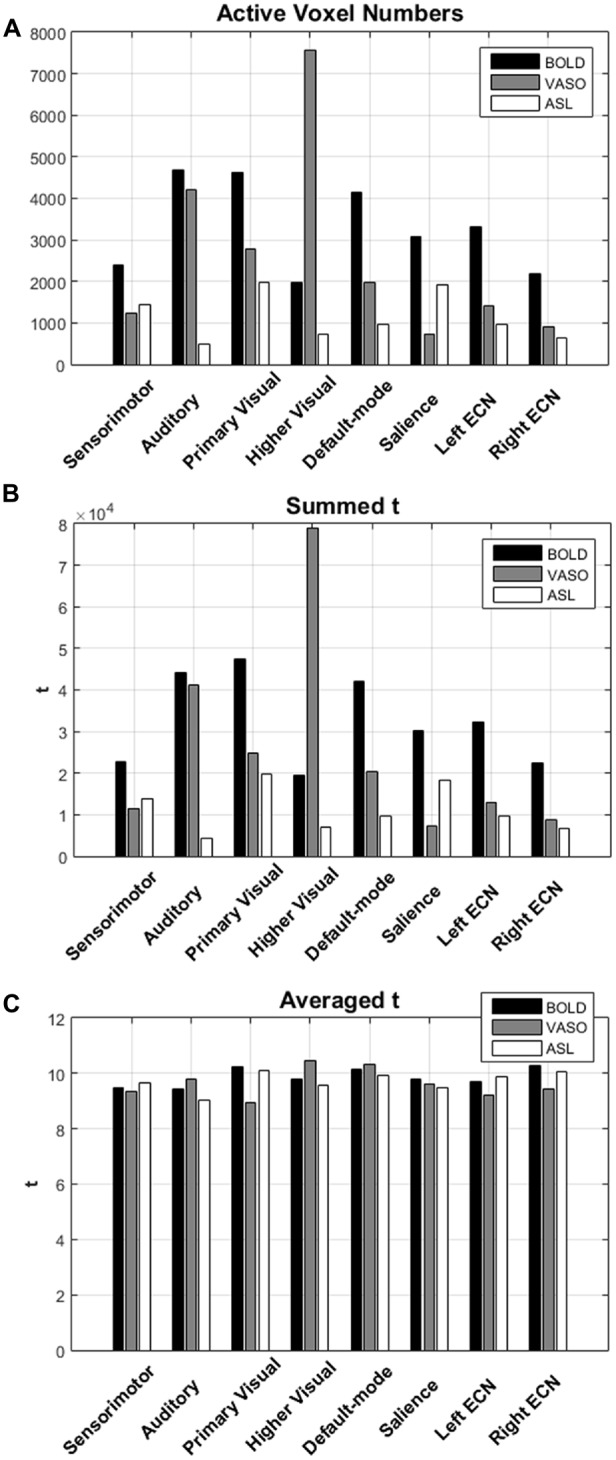
**(A)** The numbers of active voxels with *t* value >7.68 in BOLD, VASO and ASL network. **(B)** The summed *t* and **(C)** averaged *t* of these active voxels in different networks.

**Figure 3:**
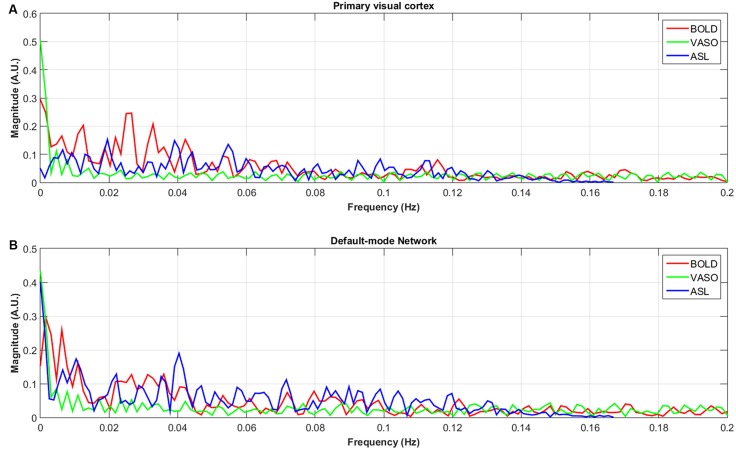
Averaged spectra of unfiltered BOLD, VASO and ASL signals in the default-node network **(B)** and primary visual cortex **(A)** of all subjects.

**Figure 4:**
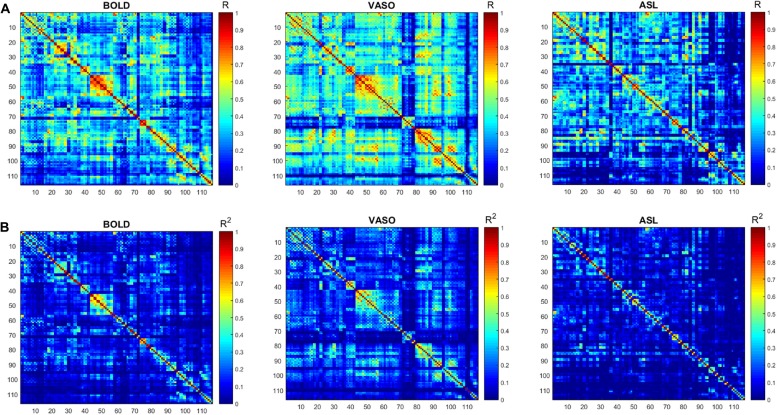
**(A)** Functional connectivity (FC) matrices (correlation coefficient *R*) derived from the BOLD, VASO and ASL. **(B)** The respective *R*^2^ matrices. Similar networks (red box) are identified by the three contrasts.

The numbers of active voxels with a *t*-value >7.68 in BOLD, VASO and ASL network maps are presented in Figure 2A. The summed t of these active voxels is presented in Figure 2B and the averaged *t* (summed *t*/number of active voxels) is presented in Figure 2C. It can be observed that in most of the networks, again with the exception of the higher visual network, BOLD has the highest number of active voxels, and the summed *t-scores*, VASO remains in the middle and ASL has the lowest values. In the sensorimotor network and salience network, BOLD has the highest active voxel number and summed *t-scores*, ASL stays in the middle and VASO has the lowest value. In the higher visual network, VASO has the highest active voxel number and summed *t-scores* while ASL has the lowest value. Averaged *t-scores* show similar values between different contrasts. Figure 3 shows the averaged spectra from all subjects. The frequency domain representation of the time courses shows that the spectral energy of BOLD fluctuation mostly falls into the low-frequency range from 0.01 Hz to 0.04 Hz. However, the spectral energy of ASL fluctuation shows more high-frequency oscillations in the default mode network. In addition to the low frequency, the high-frequency range of the ASL fluctuation is between 0.08 Hz and 0.16 Hz in the default mode network. Since the fluctuation components of BOLD, and although CBF signals are well separated in the frequency domain, BOLD contamination in the CBF signal can be suppressed by a high-pass filtering of the ASL data. As shown in Figure 4, similar networks can be identified in the correlation matrices. The functional networks around AAL regions, i.e., region number 12 (Frontal_Inf_Oper_R), 27 (Rectus_L), 40 (ParaHippocampal_R), 50 (Occipital_Sup_R), 60 (Parietal_Sup_R), 75 (Pallidum_L), 80 (Heschl_R), are found to be significant using the *t*-test (*p* < 0.05) in all contrasts (Figure 4).

## Discussion

The measurement of FC in the brain has attracted tremendous interest in the neurosciences as well as in clinical practice. For this purpose, BOLD imaging has been widely used in resting-state fMRI studies. BOLD has high sensitivity and is easy to implement. However, BOLD signal fluctuations represent combined changes in blood oxygenation, CBV, CBF and CMRO_2_, making it challenging to interpret the BOLD signal (Davis et al., 1998; Hoge et al., 1999; Buxton et al., 2004). Furthermore, CBV and CBF-based signals provide better spatial localization than the BOLD signal (Jin and Kima, 2008; Donahue et al., 2009). Therefore, imaging of less-confounding physiological parameters, such as CBV or CBF, is needed for further investigation.

In this work, resting-state brain activation detected using BOLD, CBV and CBF were compared. The spontaneous fluctuation of the CBV-weighted signal was measured using whole-brain 3D GRASE based VASO imaging. Compared to GE-EPI BOLD, the 3D-GRASE sequence used for VASO has a comparable temporal resolution, higher SNR and is less sensitive to susceptibility-induced image distortions. Quantitative CBF was measured using a 2D EPI-based pCASL sequence. High perfusion sensitivity was achieved by using a long labeling pulse. Subtraction of surrounding tissue was used to achieve a similar temporal resolution to BOLD. High-pass filtering was added into the post processing of CBF to suppress BOLD fluctuations.

**Figure 5:**
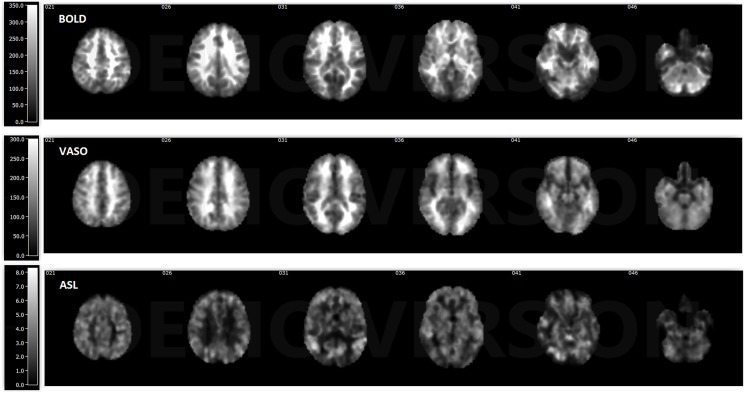
Temporal signal-to-noise ratio (tSNR) maps of BOLD, VASO and cerebral blood flow (CBF) based ASL from a representative subject. BOLD has the highest tSNR at the range of 0–350, the tSNR of VASO is in the range of 0–300 and ASL has the lowest tSNR in the range of 0–8.

**Figure 6:**
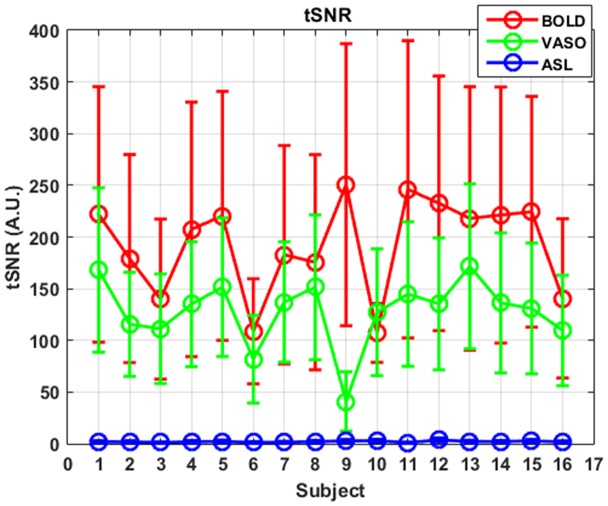
tSNR curves of three different contrasts along subjects. Mean values are marked with circles and standard deviations are shown as error bars.

ASL usually has reduced sensitivity to neural activity compared to BOLD due to the lower SNR (Viviani et al., 2011; Chen et al., 2015). In this study, VASO has been shown to be more sensitive than ASL and less sensitive than BOLD in most of the networks. One reason behind this phenomenon is the lower SNR caused by the subtraction of images in ASL. In ASL, the perfusion contrast comes from the subtraction of successively acquired images: one with and one without the labeling of arterial spins after a delay time. The subtracted signal is on the order of 1% of the baseline signal, and the resting-state fluctuations cause merely an additional fractional change. After comparing temporal SNR (tSNR) with BOLD, VASO and ASL, it was found that BOLD has the highest tSNR (in the range of 0–350), VASO is in the middle (in the range of 0–300) and the CBF of ASL has the lowest tSNR (in the range of 0–8), as presented in Figures 5, 6. The order of tSNR scores coincides with the order of the sensitivity of the functional activity in the resting-state from each contrast-based measurement. ASL has the added advantage that CBF is fully quantitative, while VASO and BOLD are not.

The seed-based analysis of the BOLD, VASO and ASL resting-state data produced similar network maps (Figure 1). Differences mainly exist in the higher visual network of VASO and the sensorimotor network, and also in the salience network resulting from ASL. CBF connectivity was found to be less extensively distributed than BOLD connectivity, which supports the findings of previous studies (Viviani et al., 2011; Chen et al., 2015). According to the number of active voxels and summed *t*-scores at *t* > 7.68, ASL shows higher values in the sensorimotor network and the salience network than in VASO. This suggests that CBF-based measurements may provide better sensitivity for detecting resting-state functional activity than VASO in these networks. One reason behind these phenomena is the short post-labeling delay (PLD) used in the ASL sequence. Although a long delay is favorable to obtain more accurate values for white matter CBF, it leads to a lower SNR owing to the magnetization decay. Higher SNR due to the short PLD increases the sensitivity of ASL in detecting the resting state networks. A short PLD will underestimate the perfusion in regions with long blood arrival time (BAT), such as white matter. Long PLD can ensure that the majority of the voxels are filled with blood but leads to decreased SNR due to signal decay (Zhang et al., 2014). With a short PLD, there is a significant large-vessel contribution to the functional CBF (fCBF) signal (Gonzalez-At et al., 2000; Zappe et al., 2008). At lower resolutions (lower than 1 × 1 × 2 mm), a shorter PLD can lead to increased *t*-values. At high resolutions, a long PLD is needed to decrease the fCBF arising from vessels. The BAT in the above networks could be lower than in other networks. In future studies, the PLD needs to be carefully decided based on the BAT in the ROIs to be accessed.

In comparison to BOLD, VASO active regions are more confined, partially due to reduced contributions from large vessels (Magnuson et al., 2010; Kim and Ogawa, 2012). The 3D-GRASE VASO sequence is intrinsically spin-echo weighted with a short TE. Compared to the gradient-echo readout, the spin-echo signal sensitivity is significantly reduced due to the refocusing of the dephasing effect around large vessels. Both BOLD and ASL techniques in this study are based on GE EPI readouts. GE BOLD sensitivity for FC is larger compared to SE BOLD, and has been demonstrated in a small number of studies (Bandettini et al., 1994; Chiacchiaretta and Ferretti, 2015). T_2_ weighted SE sequences have been proposed as an interesting alternative when increased functional localization to the capillary bed is desired. This is because static dephasing effects around larger vessels are refocused by the 180° radiofrequency pulse, trading sensitivity for a higher spatial specificity for the microvasculature (Norris, 2012). Furthermore, the sensitivity of VASO is even lower due to the decreased SNR caused by the inversion pulse. Although 3D-GRASE VASO has lower sensitivity compared to BOLD, it exhibits less susceptibility distortion in the air-tissue regions such as those around the nasal sinuses, orbital frontal lobe and temporal lobe (Figure 5). SE EPI may, therefore, provide an interesting alternative for fMRI in regions affected by macroscopic magnetic field inhomogeneities (Schwarzbauer et al., 2010; Chiacchiaretta and Ferretti, 2015; Khatamian et al., 2016). In the future, the same readout for the three modalities should be chosen for a fair comparison. For VASO, a 3D readout, such as 3D fast GRE (Cheng et al., 2017), is needed for whole brain coverage.

It is also possible that the dynamics of spontaneous fluctuations in CBF, CBV and BOLD differ depending on their location within the brain. In other words, many of these network differences could be biologically driven rather than being driven by the specifics of the selected measurement technique.

One limitation of this study is that no respiratory, cardiac, or pulse oxygenation monitoring took place during scanning, and thus no RETROICOR type physiological signal correction was possible *post hoc*.

For FC analysis, a seed-based approach was applied in this study instead of independent component analysis (ICA). ICA decomposes fMRI data into a set of independent spatial maps and associated time courses and has the advantage of being a data-driven technique. However, although similar resting state networks can be obtained with either of the two analysis methods (Van Dijk et al., 2010), recent studies suggest that the seed-based method offers greater reliability and reproducibility of connectivity measures at the individual subject level (Franco et al., 2013). Since there is still a possibility that CBF-specific or CBV-specific networks could emerge from the data if a novel seed location were used, “negative controls” need to be performed. Networks from brain locations known to play no part in any network at all were extracted. The results from this type of negative control (Supplementary Figure S1) show no recognized network in all three modalities.

Except for the fluctuations in the low-frequency range from 0.01 Hz to 0.04 Hz, the spectral energy of ASL fluctuations in the default mode network show more high-frequency oscillations. These results are consistent with previous studies (Chuang et al., 2008; Miao et al., 2014). Unfiltered resting state ASL and BOLD spectra show well separated BOLD (<0.08 Hz) and CBF (>0.08 Hz) fluctuations in the frequency domain (Chuang et al., 2008), suggesting that BOLD contamination in the CBF signal can be suppressed by high-pass filtering the ASL data.

The correlation matrices of BOLD, VASO and ASL show a similar distribution of different networks. Further topological analysis of function networks can be performed based on these correlation matrices (Stam and Reijneveld, 2007; Melie-García et al., 2013; Liang et al., 2014).

## Conclusion

This study demonstrates a comparison of resting-state brain activation detected by three different contrasts, including BOLD, CBV and CBF. The non-invasive and non-contrast agent methods of 3D-GRASE based VASO and pCASL were used to obtain separate CBV and CBF signals. Reliable brain networks were detected using VASO and ASL, and included the sensorimotor, the auditory, the primary visual, the higher visual, the default mode, the salience and the left/right executive control networks. Differences between the resting-state activation detected by ASL, VASO and BOLD might be technique-dependent due to the different tSNR, the short PLD in ASL and the spin-echo readout of VASO. It is also possible that the dynamics of spontaneous fluctuations in BOLD, CBV and CBF are biologically driven and differ depending on their location within the brain.

## Author Contributions

KZ designed the work and drafted the article. KZ and DH collected the data, performed the data analysis and interpretation. DH and NS provided critical revision of the article. All authors approved the final version to be published.

## Conflict of Interest Statement

The authors declare that the research was conducted in the absence of any commercial or financial relationships that could be construed as a potential conflict of interest.

## Footnotes

^1^http://rfmri.org/DPARSF
