# Supplementary material for: Comparison of Resting-State Brain Activation Detected by BOLD, Blood Volume and Blood Flow
Source: Front Hum Neurosci. 2018 Nov 8;12:443. doi: 10.3389/fnhum.2018.00443 (PMC6235966; doi:10.3389/fnhum.2018.00443)
Supplement: Supplementary file 1 [file Data_Sheet_1.docx]

Supplementary Material

Comparison of resting-state brain activities detected by BOLD, blood volume and blood flow

Ke Zhang, Dengfeng Huang, N. Jon Shah*

*** Correspondence:** N. Jon Shah: n.j.shah@fz-juelich.de

## Supplementary Figures


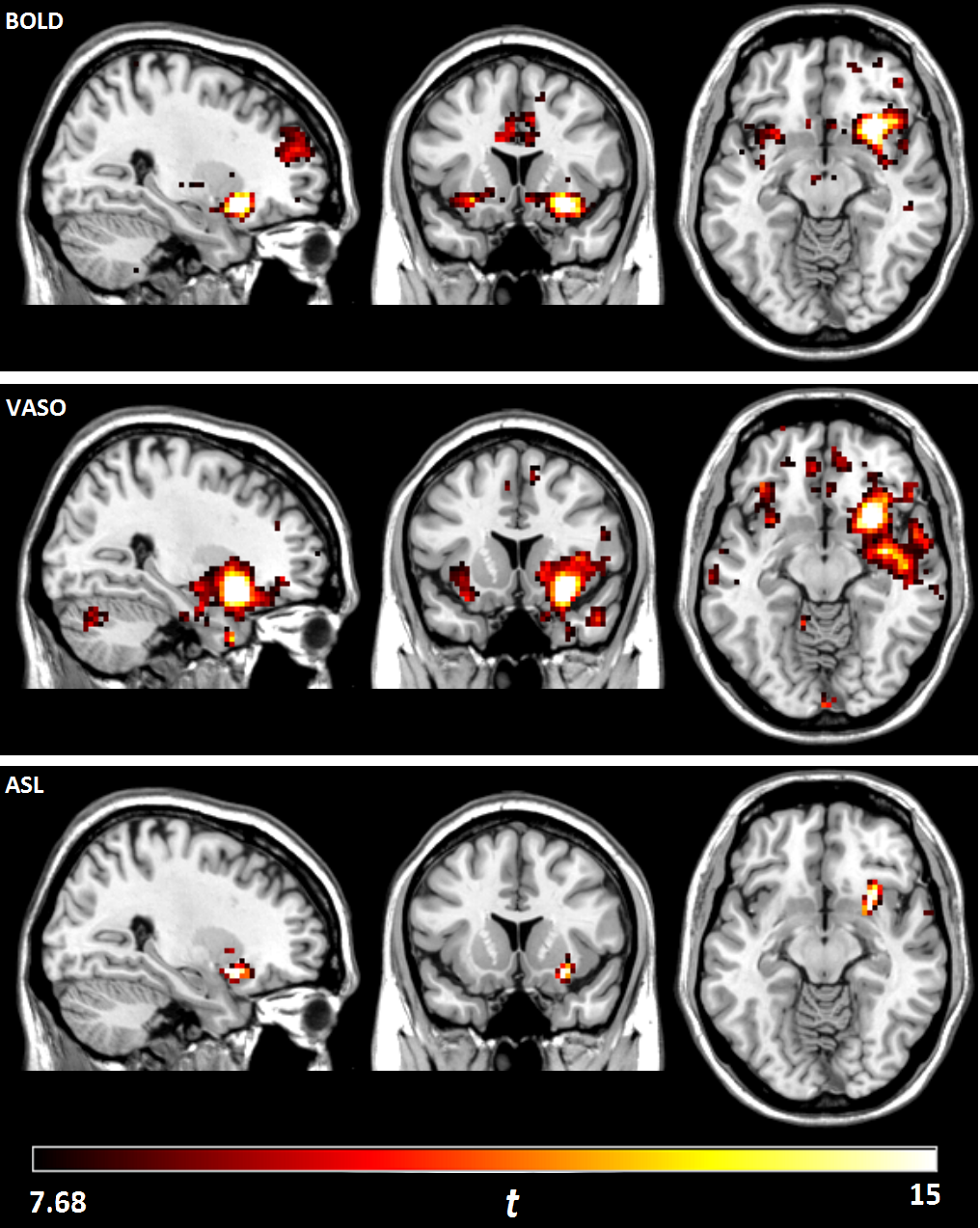


**Supplementary Figure 1.** One seed at the location near right putamen (MNI coordinate: 27, 14, -11) was placed and networks were extracted from all 3 modalities. No recognized network was found from this negative control.
